# Collectively coping with coronavirus: Local community identification predicts giving support and lockdown adherence during the COVID‐19 pandemic

**DOI:** 10.1111/bjso.12457

**Published:** 2021-05-10

**Authors:** Clifford Stevenson, Juliet R. H. Wakefield, Isabelle Felsner, John Drury, Sebastiano Costa

**Affiliations:** ^1^ Nottingham Trent University UK; ^2^ University of Sussex UK; ^3^ Università Degli Studi della Campania Luigi Vanvitelli Naples Italy

**Keywords:** community, COVID‐19, helping, norms, social identity, social support

## Abstract

The role of shared identity in predicting both ingroup helping behaviour and adherence to protective norms during COVID‐19 has been extensively theorized, but remains largely under‐investigated. We build upon previous Social Identity research into community resilience by testing the role of pre‐existing local community (or ‘neighbourhood’) identity as a predictor of these outcomes, via the mediator of perceived social support. Community residents in the UK completed a longitudinal online survey four months before lockdown (T1; *N* = 253), one month before lockdown (T2; *N* = 217), and two months into lockdown (T3; *N* = 149). The cross‐lagged panel analysis shows that T1 community identification predicts T3 giving and receiving of pandemic‐related support, and that these effects occur via the perception of community support at the second time point (while the alternative pathway from T1 support via T2 identification is non‐significant). Moreover, we show that T1 community identification also directly predicts lockdown adherence at T3. Our findings point to the pivotal role played by community identity in effective behavioural responses to the pandemic, and the need to support and foster community development to facilitate local community resilience as the crisis continues to unfold.

## Background

The UK was exceptionally badly affected by the first wave of the COVID‐19 global pandemic, suffering over 290,504 cases of infection and 44,883 officially recorded deaths by 13 July 2020 (Johns Hopkins Coronavirus Resource Centre, 2020). In response to rapidly escalating infections, a lockdown was introduced on 23 March 2020 and lasted for 14 weeks in various forms, before an easing of many of the restrictions across most areas of the UK in early July. During this time, the entire population (except for key‐workers) was instructed to ‘stay at home’, and could only leave for one time‐limited exercise break per day unless needing to obtain food or medicine, provide care for the sick or work in an essential occupation (where working from home was not possible). Over time, this strategy proved effective, with a reduction in infection rates to the point where the Government decided to introduce a series of relaxations of the restrictions in June and July.

While ‘staying at home’ demonstrably reduces the rate of infection (Hale et al., 2020, 2020), it also incurred a heavy toll on the health and well‐being of the public. Many socially and economically vulnerable groups suffered excessively, with 48% of households incurring hardship as a result of the restrictions on their movement and employment. Nonetheless, levels of adherence to the rules on lockdown were very high, on both behavioural and self‐report measures, even among groups less vulnerable to the effects of the disease (Aguilar‐Garcia, 2020; Jackson et al., 2020).

Among those most severely affected by lockdown were older and medically vulnerable adults who required urgent assistance to meet their basic needs for food and medical supplies. Often, especially in the early stages of the pandemic, this need went unmet by local health and social care services, due to them being overwhelmed by demand. In most areas across the UK, local residents’ associations, often under the umbrella of ‘COVID‐19 Mutual‐Aid Groups’, stepped in to provide basic assistance to the most vulnerable of their residents (Booth, 2020; Hogan, 2020; Stansfeld, Mapplethorpe, & South, 2020).

Given the importance of public engagement in an effective response to COVID‐19 – both in terms of observing lockdown and in terms of mutual aid among neighbours – a key question for theory and policy is that of the determinants of these behaviours. Recent research suggests that group‐level perception is pivotal to effective crisis response (e.g. Biddlestone, Green, & Douglas, 2020; Goldberg et al., 2020, 2020). However, few researchers have examined the actual psychological processes underlying these results, and none have captured the unfolding community identity dynamics underpinning local residents’ responses to the crisis. In the present paper, we report a three‐wave longitudinal community survey that examines the extent to which community identity predicts lockdown adherence and the exchange of assistance via the perceived normativity of social support in the community.

### The social identity approach to public behaviour in the coronavirus pandemic

In attempting to theorize the psychological underpinnings of both helping behaviour and norm adherence during the current pandemic, social psychologists have pointed to the key role played by group processes (Van Bavel et al., 2020; Jetten, Reicher, Haslam, & Cruwys, 2020). Social Identity theorists in particular have highlighted the inherently collective nature of the experience of the crisis – the need for collective adherence to norms of infection‐reducing behaviour, and the evidence of a collective behavioural response to the crisis – as indicating the irreducibly group‐level nature of the current situation (Drury, Reicher, & Stott, 2020; Jetten et al., 2020). Following basic tenets of self‐categorization theory (Turner, Hogg, Oakes, Reicher, & Wetherell, 1987), they argue that the sharing of identity within a group unlocks intragroup dynamics of help and support exchange, social influence, and collective action (Haslam, Jetten, Cruwys, Dingle, & Haslam, 2018). Shared identity increases trust, supportive behaviour and reciprocal helping between ingroup members, which in turn displays or ‘models’ selfless behaviour to others (Drury, 2018). Such displays are likely to influence fellow ingroup members, who will be inclined to internalize these norms of acting in the public good, which then become further established across the group. A shared identity‐related norm of mutual helping also provides a platform whereby the group can be shaped and mobilized towards collective action to protect fellow members (Drury, Brown, González, & Miranda, 2016).

Shared identity also predicts the *receipt* of support from others: the bonds of trust and reciprocity created by a sense of belonging to a group characterized by helping encourages those who are offered support to accept it in the positive spirit in which it was intended, rather than with suspicion or discomfort, and to perceive the support as being effective at meeting their needs (Haslam et al., 2018). Understanding these processes is vital not only to explain and predict how people are behaving during the pandemic, but to identify ways of encouraging and promoting forms of group behaviour which can enable the public to deal more effectively with this challenge.

Certainly, a broad range of Social Identity research prior to the current crisis supports this contention. In particular, the study of collective responses to emergencies and disasters has established that the collective social identity emerging from such events leads to helping: rather than engaging in selfish and individualistic behaviours, group members express solidarity through cooperation and collective helping, as evident in the aftermath of the July 7th 2005 bombings in London (Drury, Cocking, & Reicher, 2009). Second, the enactment of these behaviours provides visible evidence to fellow group members of the availability of support when it is required. This leads to a greater sense of personal and collective efficacy, as well as the potential of the group to act together in concert (Drury et al., 2016). Third, these factors then feed forward into coordinated action, allowing people to cope collectively with the emergency. On this basis, we would expect that sharing a community identity would lead to adherence to group‐protective norms, while the social support flowing from should lead in turn to the giving and receipt of help during a crisis.

Early empirical studies of the public response to the crisis are certainly supportive of the applicability of this theoretical perspective. National, political and family memberships have all been implicated in the experience of and response to the current situation (Prime, Wade, & Browne, 2020; Rothgerber et al., 2020; Sibley et al., 2020). Likewise, several longstanding psychological attributes including collectivist orientation have been shown to predict prosocial behaviour during the crisis. For example, Biddlestone et al., (2020) found that collectivistic (rather than individualistic) thinking is a better predictor of observance of ‘social distancing’, while Goldberg et al., (2020, 2020) demonstrate how the belief that friends and family are engaging in disease prevention behaviours predicts successful adherence to these behaviours. Moreover, the role of social support has already been demonstrated to be pivotal to the ability of individuals to cope with the negative mental health impacts of the crisis (Bauer et al., 2020) and the receipt of help from others in particular has been found to be associated with adherence to disease preventative norms (Smith et al., 2020).

However, while this array of findings is certainly consonant with a group‐level understanding of the crisis, none have captured the unfolding social identity dynamics of collective responses. Moreover, this research has yet to directly examine the local community identities to which help‐giving and norm adherence have typically been attributed. Templeton and colleagues (Templeton et al., 2020) have drawn attention to this gap, arguing that the common fate shared by local communities should serve as the basis for an emergent response to the virus, something that may be undermined by local community inequality or division. However, researchers have yet to investigate the particular role played by the local community or ‘neighbourhood’ in providing the platform of shared social identity required for this collective engagement.

### Neighbourhood identity and intragroup solidarity

The ability of local neighbourhoods to provide support to their residents is well‐established across the social sciences. Studies of ‘social capital’ have shown that the associative behaviour of neighbours has substantial impacts on the health and well‐being of community members (Ehsan, Klaas, Bastianen, & Spini, 2019; Pretty, Bishop, Fisher, & Sonn, 2006). The way in which social capital is thought to impact upon well‐being is through the giving and receiving of help (Ehsan et al., 2019). The social support received from one’s fellow community members has direct practical benefits (Perkins & Long, 2002; Poortinga, 2006), but also serves to increase feelings of belonging, and reduce the negative effects of loneliness (Wakefield et al., 2020). The benefits of giving help derive from the impacts upon self‐worth and self‐efficacy, but also from the enhanced closeness to neighbours (Bowe et al., 2020; Pretty et al., 2006).

This giving and receiving of help between neighbours also serves to build up norms of trust and reciprocity (Putnam, 2000). Residents learn to depend upon the goodwill of their neighbours on a day‐to‐day basis, as well as knowing that they will be able to depend upon them in crisis. Where these norms are well‐established, they predict a local community’s ability to endure and recover from unexpected challenges, including human‐made and natural hazards (Aldrich, 2012). In other words, the perception of the availability of support in local neighbourhoods will impact directly upon helping behaviour during future threats, predictions borne out by early reports of the higher levels of mutual aid groups in areas of high social capital during the current crisis (Felici, 2020; Tiratelli & Kaye, 2020) and the association between social capital and adherence to ‘social distancing’ measures (Sharkey, 2020).

Recently, social psychologists working within the Social Identity Approach to Health (otherwise known as the ‘Social Cure’ perspective; Haslam et al., 2018) have provided a more coherent theoretical explanation for why this relationship between community membership and resilience may occur. Neighbourhoods, they argue, are an important social group for most people, as they form the immediate context for much of their daily lives, as well as providing an ever‐present, and often influential, cohort of peers (Fong, Cruwys, Haslam, & Haslam, 2019a; Stevenson et al., 2019). The benefits of neighbourhood for well‐being accrue when residents identify (i.e. experience a subjective sense of commonality with) with their neighbours, which unlocks a range of collective social and psychological processes, including the willingness and ability to act in concert with fellow residents to deal with shared challenges and increased perceptions of trust and support.

Empirical work has shown that neighbourhood identification is indeed associated with higher well‐being among residents of deprived urban neighbourhoods (McNamara, Stevenson, & Muldoon, 2013). Likewise, neighbourhood identification buffers the effects of low neighbourhood socioeconomic status on the mental health of residents (Fong et al., 2019a), provides them with resilience to cope with the effects of gentrification (Fong, Cruwys, Haslam, & Haslam, 2019b) and moderates the impact of financial stress upon residents’ well‐being (Elahi et al., 2018). One way in which neighbourhood has its well‐being benefits is through the provision of social support. Local community identification promotes the perception of the availability of social support from neighbours, which is associated with improved well‐being, as well as an enhanced ability to deal with the challenges posed by neighbourhood demographic change (Stevenson, Costa, Easterbrook, McNamara, & Kellezi, 2020; Stevenson et al., 2019). Additionally, qualitative research has shown that perceptions of social support serve as a signifier for acceptance of newcomers within newly diversified neighbourhoods, promoting residents’ sense of belonging, their willingness to give and receive help and their ability to act with others to preserve community cohesion (Stevenson & Sagherian‐Dickey, 2016). Overall, there is considerable evidence that neighbourhood identification provides residents with the psychological resilience needed to deal with future challenges.

### The current study

In sum, the Social Identity approach posits that shared identity dynamics have underpinned the public response to the coronavirus by facilitating the supportive and protective behaviour widely documented in communities, both across the UK and internationally. This fits well with the recent Social Cure investigations of the role of local community identity in providing psychological and social resilience to local residents facing marginalization and threat. However, research has yet to determine whether community identity predicts residents’ adaptive response to the virus, and, if so, how this occurs.

In the current study, we therefore use a longitudinal survey method to establish the degree to which pre‐existing community identification predicts giving and receipt of emotional support during lockdown, as well as their adherence to lockdown norms intended to reduce COVID‐19’s spread. Moreover, we aim to identify the processes underpinning these relationships by exploring whether they are mediated by pre‐existing perceived community support. We therefore predict that:

Hypothesis 1 In line with the Social Cure model, community identification at Time 1 (T1: pre‐lockdown) will positively predict adherence to group‐supportive norms in the form of rules designed to halt virus spread within the community at Time 3 (T3).

Hypothesis 2 Community identification at Time 1 (T1: pre‐lockdown) will positively predict the giving (H2a) and receiving (H2b) of emotional support under lockdown at Time 3 (T3).

Hypothesis 3 As community identity is theorized to have effects on supportive behaviour through increased intragroup trust and the expectation of reciprocal helping, the relationship between T1 community identification and T3 giving (H3a) and receiving (H3b) of emotional support will be mediated by the presence of perceived community support at Time 2 (T2: pre‐lockdown).

## Method

### Participants and procedure

Two‐hundred and sixty‐four UK‐residing adult participants (170 females, 87 males, 7 other; *M*_age_ = 35.73 years, *SD* = 12.90, *range* = 18–71) were recruited via Prolific Academic, and completed the first wave of a three‐wave online survey in November 2019.^1^ Participants were paid £2 on completion of the survey. Eleven participants were removed from the data file because they completed too little of the survey to produce analysable results. This led to a time 1 (T1) sample of 253 (162 females, 86 males, 5 other; *M*_age_ = 35.45 years, *SD* = 12.50, *range* = 18–71).

Of these who provided information, 171 (69%) were employed, 31 (13%) were unemployed, 16 (6%) were retired, and 30 (12%) were students. In terms of monthly income after tax, 26% made < £999, 38% made £1,000–£1,999 and 36% made £2,000 or more. Finally, in terms of education, 55% had an undergraduate or postgraduate degree.

Three months later (*M* = 94.52 days, *SD* = 4.73, *range* = 91.91–113.07 days), participants completed the study’s second wave, in February 2020. Participants were paid £1.50 on completion of the survey (less than at T1, due to the T2 survey being shorter). Two‐hundred and twenty‐six participants responded, but data for 9 participants were removed from the data file because they completed too little of the survey to produce analysable results. This led to a time 2 (T2) sample of 217 (144 females, 69 males, 4 other; *M*_age_ = 36.06 years, *SD* = 12.73, *range* = 18–71).

Three months later (*M* = 89.79 days, *SD* = 5.49, *range* = 69.66–112.68 days), participants completed the study’s third wave, in May 2020. Participants were paid £1.70 on completion of the survey (more than at T2 due to the additional pandemic‐related items). One‐hundred and seventy‐seven participants responded, but data for 28 participants were removed from the data file because they completed too little of the survey to produce analysable results, or they had moved house between time‐points. This led to a time 3 (T3) sample of 149 (100 females, 45 males, 4 other; *M*_age_ = 37.64 years, *SD* = 12.61, *range* = 18‐71). Due to the COVID‐19 pandemic (and the associated lockdown in the UK) occurring shortly after T2, the COVID‐19‐related items (described later) were only included in the T3 survey.

An analysis of variance was conducted to compare the T1 participants who did vs. did not complete the T2 survey. These groups did not differ significantly in terms of age, community identification, or community support (*p*s ranged from .06 to .71). A chi‐square analysis was also conducted, which revealed that the groups differed significantly in terms of the number of males and females, *X^2^* (3) = 10.14, *p* = .02, with males making up 47% of non‐responders, but only 32% of responders. Nonetheless, based on these analyses, it was concluded that the participants who completed the T3 survey were a reasonable representation of the sample as a whole.

An analysis of variance was conducted to compare the T1 participants who did vs. did not complete the T3 survey. These groups did not differ significantly in terms of community identification or community support (*p*s ranged from .19 to .88), but participants who completed the T3 survey were significantly older than participants who did not, *F*(1, 251) = 11.69, *p* = .001. A chi‐square analysis was also conducted, which revealed that the groups did not differ significantly in terms of the number of males vs. females (*p* = .10). Based on these analyses, it was concluded that the participants who completed the T3 survey were a reasonable representation of the sample as a whole.

### Measures

#### Community variables

*Community identification* was measured with Doosje, Ellemers, and Spears’ (1995) four‐item Group Identification Scale. Participants rated their agreement with each item (e.g. ‘I see myself as a member of my local community’) on a scale ranging from 1 (‘I strongly disagree’) to 7 (‘I strongly agree’). The mean of the items was found, with higher values indicating higher levels of community identification. The group‐level intraclass correlation coefficient (ICC) was .84 between T1 and T2, .83 between T1 and T3, and.87 between T2 and T3, which are above the ‘excellent’ reliability cut‐off of .75 (Fleiss, 1986).

*Perceived Community Support* was measured with Haslam, O'Brien, Jetten, Vormedal, and Penna (2005) Social Support Scale. Participants rated their agreement with each item (e.g. ‘Do you get the emotional support you need from other people in your local area?’) on a scale ranging from 1 (‘I strongly disagree’) to 7 (‘I strongly agree’). The mean of the items was found, with higher values indicating higher levels of perceived community support. The group‐level intraclass correlation coefficient (ICC) was .81 between T1 and T2, .83 between T1 and T3, and .79 between T2 and T3.

*Demographic variables* were also gathered, which included the participants’ *age* and *gender*. These were conceptualized as control variables.

#### COVID‐19 variables

As mentioned above, the following items were presented in the T3 survey only. Participants’ *giving of emotional support during the pandemic* was measured with an adapted version of Drury et al., and and’s (2016) three‐item Provided Emotional Social Support Scale. Participants were asked to think about the previous three months and to rate the frequency with which they had engaged in each behaviour in response to COVID‐19 (‘Gave emotional support’; ‘Showed respect for others’; and ‘Showed concern for others’ needs’), using a scale ranging from 1 (‘Not at all’) to 5 (‘To a very great extent’). The mean of the items was found, with higher values indicating higher levels of given emotional support.

Participants’ *receipt of emotional support during the pandemic* was measured with an adapted version of Drury et al., and and’s (2016) three‐item Provided Emotional Social Support Scale. Participants were asked to think about the previous three months, and to rate the extent to which others had engaged in each behaviour towards the participant in response to COVID‐19 (‘Gave you emotional support’; ‘Showed respect for you’; and ‘Showed concern for your needs’), using a scale ranging from 1 (‘Not at all’) to 5 (‘To a very great extent’). The mean of the items was found, with higher values indicating higher levels of received emotional support.

Participants’ *adherence to lockdown rules* was measured with a single item: ‘During the past three months, to what extent have you adhered to the coronavirus lockdown rules (e.g., only leaving the house for food, medicine, daily exercise, caring for the sick, and going to work if you cannot work from home)?’. Participants indicated their adherence on a scale ranging from 1 (‘Not at all’) to 5 (‘To a very great extent’).

Participants’ *vulnerability to COVID‐19* was measured with a single item: ‘Are you categorised by your government/health service as particularly vulnerable to coronavirus (e.g., due to chronic health issues)?’ Participants responded with either *yes* or *no*. Six participants reported that they did not know whether they were vulnerable or not, so their ‘don’t know’ response was replaced with a blank value. Vulnerability was conceptualized as a control variable^1^.

## Results

### Descriptives and correlations

The descriptive statistics for the key variables (and the correlations between them) can be found in Table 1.

**Table 1:** Means, standard deviations, alphas (where appropriate), and correlations for key variables

Variable	1	2	3	4	5	6	7	8
1. Community Identification T1 (1–7, *M* = 3.98, *SD* = 1.45, *α* = .93)	–							
2. Community Identification T2 (1–7, *M* = 4.07, *SD* = 1.37, *α* = .93)	.73***	–						
3. Community Identification T3 (1–7, *M* = 4.20, *SD* = 1.36, *α* = .93)	.71***	.75***	–					
4. Perceived Community Support T1 (1–7, *M* = 3.56, *SD* = 1.50, *α* = .94)	.62***	.54***	.48***	–				
5. Perceived Community Support T2 (1–7, *M* = 3.60, *SD* = 1.50, *α* = .94)	.59***	.72***	.57***	.68***	–			
6. Perceived Community Support T3 (1–5, *M* = 3.74, *SD* = 1.49, *α* = .94)	.53***	.46***	.60***	.71***	.66***	–		
7. Giving Pandemic Support T3 (1–5, *M* = 2.85, *SD* = .88, *α* = .76)	.31***	.47***	.42***	.37***	.39***	.39***	–	
8. Receiving Pandemic Support T3 (1–5, *M* = 2.47, *SD* = 1.04, *α* = .88)	.42***	.52***	.42***	.53***	.51***	.57***	66***	–
9. Lockdown Adherence T3 (1–5, *M* = 4.55, *SD* = .76)	.22**	.27**	.38***	.05	.17*	.15^†^	.28**	.09

Note

Values have been computed with all available data (so, *n* = 253 for T1 variables, *n* = 217 for T2 variables, and *n* = 149 for T3 variables).

*** *p* < .001,

** *p* < .01,

* *p* < .05,

^†^ *p* = .07.

Supporting hypothesis 1, T1 community identification correlated positively with T3 lockdown adherence (*p* = .008). Supporting hypotheses 2a and 2b, T1 community identification correlated positively with T3 giving and receiving of pandemic‐related emotional support (*p*s < .001). Providing initial support for hypotheses 3a and 3b, T2 perceived community support correlated positively with T3 giving of pandemic‐related emotional support (*p* < .001) and receiving of pandemic‐related emotional support (*p* < .001). Controlling for age, gender, and vulnerability to COVID‐19 did not affect the overall patterning of the correlations, although the correlation between T2 perceived community support and T3 lockdown adherence became non‐significant (*p* = .13).

### Cross‐lagged panel analyses

**Figure 1:**
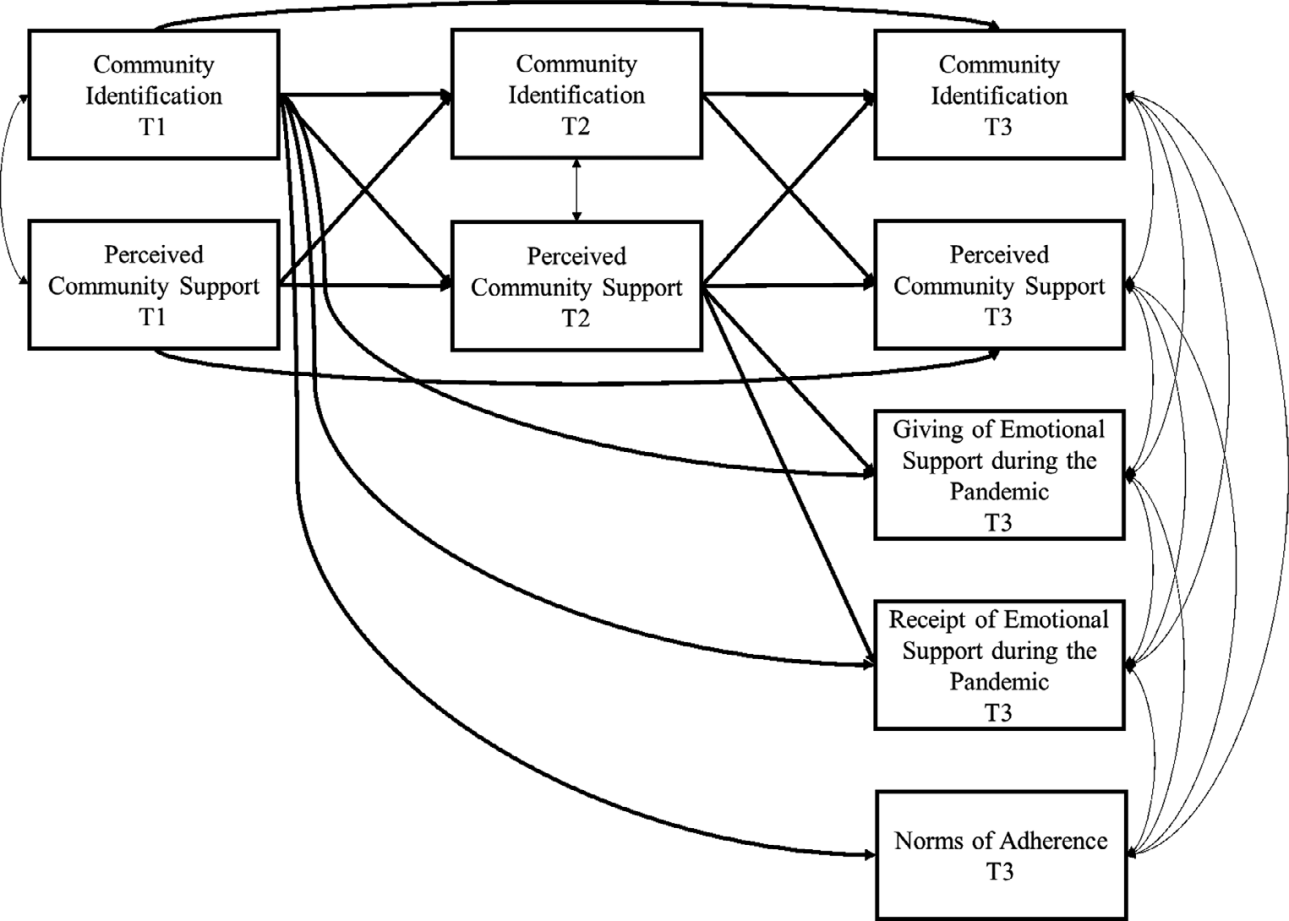
Proposed cross‐lagged panel model of T1 predictors, T2 mediators, and T3 outcomes.

A model integrating the autoregressive and cross‐lagged paths between the study variables was tested (Figure 1). Specifically, the model took into account the stability of community identification and perceived community support over time, and the within‐wave correlations between community identification and perceived community support at T1 and T2, as well as the correlations between the T3 variables (community identification, perceived community support, the giving of emotional support during the pandemic, the receipt of emotional support during the pandemic, and lockdown adherence).^2^ As the latter three of these variables were not measured pre‐pandemic, there were no T1//T2 versions to include in the analysis.

Furthermore, the bidirectional association between community identification and perceived community support over the three waves was tested, and also the direct path from T1 community identification and T2 perceived community support to T3 giving of emotional support during the pandemic, and T3 receipt of emotional support during the pandemic at T3. Finally, in line with the theoretical foundation described in the Introduction, a path from T1 community identification to T3 lockdown adherence was added. Participants’ gender, age, and vulnerability to COVID‐19 were included as control variables, which added paths from these control variables to each of the study variables (these paths are omitted from Figure 1 for clarity). The lavaan package within R (Rosseel, 2012) was used to compute the proposed model and estimate parameters, and the bootstrapping approach with 5,000 bootstrap replication samples for the coefficient intervals was used to evaluate total, direct, and indirect effects.

**Figure 2:**
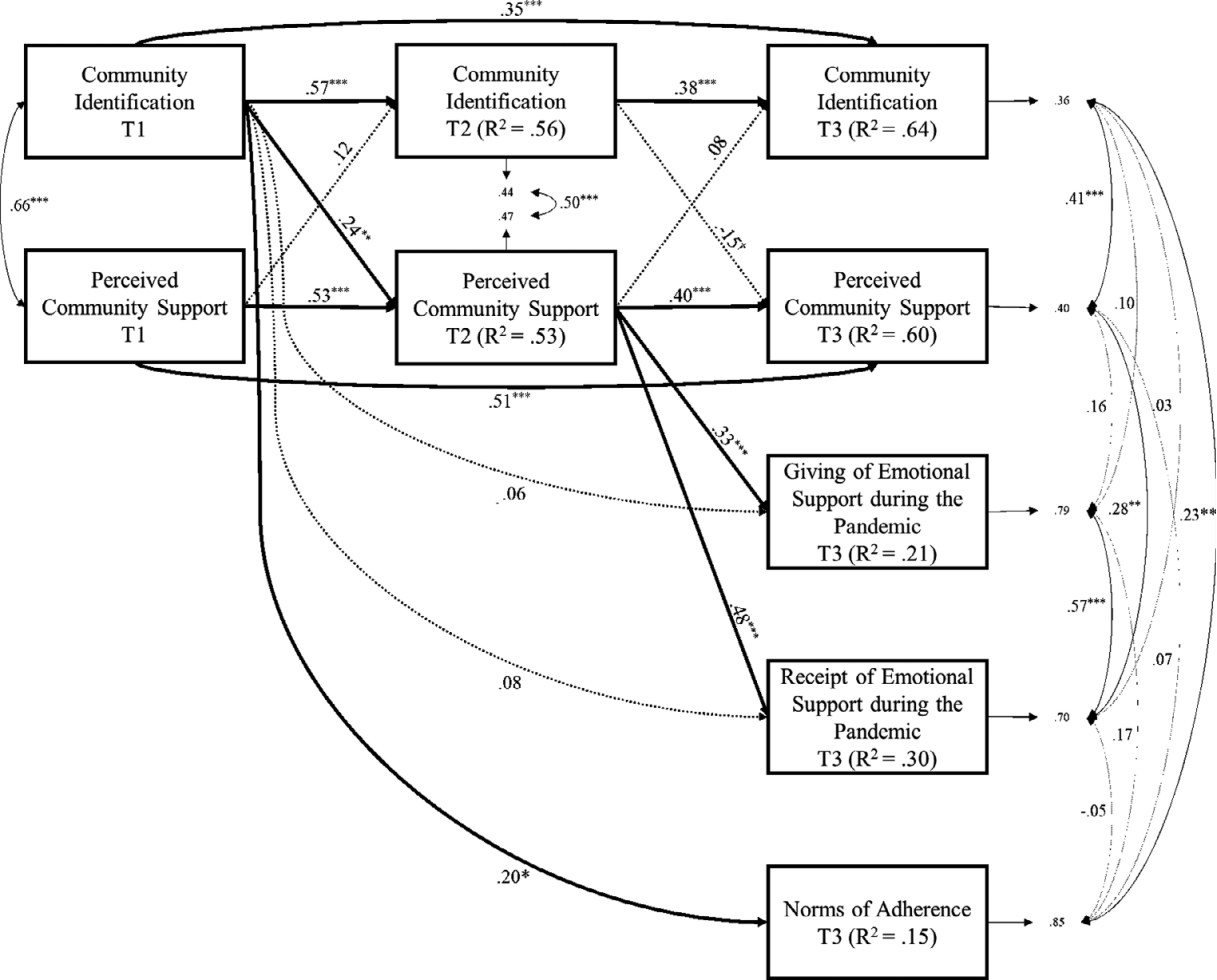
Cross‐lagged panel model of the effects of T1 predictors on T3 outcomes via T2 mediators. Participants’ age, gender, and income are included in the analysis as control variables (though not shown here for clarity). *Note*: *** *p* < .001, **p < .01, *p < .05, ^†^ *p* < .10.

Estimation of the model (Figure 2) showed a total coefficient of determination (TCD; Canale et al., 2016; Jöreskog & Sörbom, 1996) of .83 that corresponds to a correlation of *r* = .91 and could be interpreted as a large effect size according to Cohen’s criteria (1988). In addition to the total variance explained by the model, the *R* ^2^ of each endogenous variable (ranging between .15 and .64) also shows modest variance explained. The findings indicate that the hypothesized model fitted the data adequately, *χ* ^2^(9) = 32.24, *p* < .01, *CFI* = .97, *SRMR* = .03, *RMSEA* (90%) *CI* = .14 (.09, .19). For community identification, there were significant first‐order autoregressive paths from T1 to T2, *b* = 0.52, *p* < .001, *CI* (0.36, 0.68), *β* = 0.57, and from T2 to T3, *b* = 0.37, *p* < .001, *CI* (0.17, 0.57), *β* = 0.38, and a significant second‐order autoregressive path from T1 to T3, *b* = 0.32, *p* < .001, *CI* (0.15, 0.49), *β* = 0.35. Similarly, for perceived community support, there were significant first‐order autoregressive paths from T1 to T2, *b* = 0.53, *p* < .001, *CI* (0.34, 0.68), *β* = 0.53, and from T2 to T3, b = 0.40, *p* < .001, *CI* (0.19, 0.60), *β* = 0.40, and a significant second‐order autoregressive path from T1 to T3, *b* = 0.51, *p* < .001, *CI* (0.33, 0.68), *β* = 0.51.

Examination of the cross‐lagged effect showed a significant path from T1 community identification to T2 perceived community support, *b* = 0.23, *p* = .01, *CI* (0.07, 0.41), β = 0.24, while the opposite path (from T1 perceived community support to T2 community identification) was not significant, *b* = 0.11, *p* = .170, *CI* (−0.05, 0.26), *β* = 0.12. Furthermore, both the cross‐lagged effects from T2 to T3 were non‐significant: neither T2 community identification to T3 perceived community support, *b* = −0.16, *p* = .10, *CI* (−0.36, 0.03), *β* = −0.15, nor T2 perceived community support to T3 community identification, *b* = 0.07, *p* = .35, *CI* (−0.07, 0.23), *β* = 0.08. Finally, examination of the hypothesized direct effects from the predictor/mediator variables to the T3 COVID‐19‐related variables showed a significant path from T2 perceived community support to T3 giving of emotional support during the pandemic, *b* = 0.20, *p* = .001, *CI* (0.09, 0.31), β = 0.33, and to T3 receipt of emotional support during the pandemic, *b* = 0.34, *p* < .001, *CI* (0.21, 0.46), *β* = 0.48. The direct path from T1 community identification to T3 giving of emotional support during the pandemic was non‐significant, *b* = 0.03, *p* = .603, *CI* (−0.09, 0.16), *β* = 0.06, as was the direct path from T1 community identification to T3 receipt of emotional support during the pandemic, *b* = 0.06, *p* = .351, *CI* (−0.07, 0.17), *β* = 0.08. Meanwhile (and in line with H1), the direct path from T1 community identification to T3 lockdown adherence during the pandemic was significant, *b* = 0.10, *p* = .024, *CI* (0.02, 0.20), *β* = 0.20.

Overall, the total effect of T1 community identification on T3 receipt of emotional support during the pandemic was significant, *b* = 0.13, *p* = .02, *CI* (0.02, 0.25), *β* = 0.19, while the total effect of T1 community identification on T3 giving of emotional support during the pandemic was non‐significant, *b* = 0.08, *p* = .18, *CI* (−0.04, 0.20), *β* = 0.14. However, the indirect effects from T1 community identification to both of these T3 outcome variables were significant: the association from T1 community identification to T3 giving of emotional support during the pandemic through T2 perceived community support was significant, *b* = 0.05, *p* = .022, *CI* (0.01, 0.09), *β* = 0.08, as was the path from T1 community identification to T3 receipt of emotional support during the pandemic via T2 community support, *b* = 0.08, *p* = .015, *CI* (0.02, 0.15), *β* = 0.11. In other words, the analysis provides evidence for the effects of T1 community identification on these outcomes (H2a, H2b), and that these effects occur through T2 perceived community support (H3a, H3b).

## Discussion

Regulating the spread of COVID‐19 in the UK through lockdown and ‘social distancing’ measures has required enormous levels of restraint and self‐sacrifice among the general public, which has undoubtedly saved the lives of many of the most vulnerable within their local communities (Aguilar‐Garcia, 2020; Jackson et al., 2020). Likewise, the emergence of helping behaviour within communities at a scale unseen in recent decades has been of considerable benefit to those in need of assistance (Booth, 2020; Hogan, 2020; Stansfeld et al., 2020). As Jetten et al., (2020) note, these responses to the pandemic have been irreducibly collective: for them to occur, residents had to feel a sense of shared social identity, thereby unlocking processes of helping, social support, and collective action. In terms of specific identities, as Templeton et al., (2020) have pointed out, local community plays a vital role in this process. Our present study begins to address the question of how local community identity, and the group dynamics flowing from sharing this identification, has enabled residents to collectively cope with the crisis.

Our first finding is that pre‐existing community identification predicts adherence to norms of observance of lockdown restrictions. This supports the Social Cure contention that strength of identification typically predicts group‐supportive norms (Haslam et al., 2018) and accords with recent studies linking disease preventative behaviour in the current crisis to the social influence of relevant groups such as family and friends (Goldberg et al., 2020, 2020). It therefore provides evidence for the previously theorized importance of local community identity in collectively responding to the pandemic.

Second, we find that pre‐crisis community identification serves to predict individuals’ self‐reported helping behaviour during the crisis and that this occurs via increased perceived neighbourly support. This accords with research on social capital which shows that communities with dense social networks of trust and reciprocity fare better in times of crisis (Aldrich, 2012), but supports the social identity contention that it is community identification which primarily increases perceptions of support. The perception of the availability of support in turn facilitates the actual giving and receiving of support in response to shared crisis. This supports previous research in the Social Cure tradition attesting to the protective qualities of neighbourhood identification in the face of threat (Elahi et al., 2018; Fong et al., 2019a, 2019b; McNamara et al., 2013) and highlights the importance of long term community cohesion in providing collective resilience to their residents in times of crisis.

In terms of limitations to the study, it is also worth bearing in mind that the sample is a convenience one, and while it is diverse in terms of age, SES, and education, it cannot be taken to reflect the prevalence or scale of these processes across the broader population. Furthermore, norm adherence was measured using a single self‐response item which may not have fully captured the range and variability of this behaviour. Adding a further wave of the survey in which norm adherence was measured in a more extensive way would overcome this, and additionally, it would allow further exploration of the extent to which helping behaviour predicts norm adherence. Currently, as both variables were measured at the same time, our ability to explore this is limited. Similarly, the measures of giving and receiving emotional support under lockdown, as well as of norm adherence, are retrospective, self‐report, and amenable to self‐presentation bias. While we are primarily interested in the relationships between these variables rather than their objective accuracy, future research would benefit from behavioural measures. Finally, the size of the sample means that some of the more subtle relationships between group processes and COVID‐19‐related behaviour may be overlooked. Additionally, due to the small sample size, it was not possible to conduct latent variable analysis, so the current analyses were run with indicators of the constructs. Future research should incorporate larger sample sizes so that latent variable analysis can be conducted.

With these limitations in mind, the present work is the first (to our knowledge) to provide evidence on how social identities shape local community responses to COVID‐19, and the first to elucidate the protective and resilient properties of neighbourhood identities during this crisis. In using a longitudinal design and an advanced cross‐lagged panel analysis, it provides robust evidence for the strength and direction of these effects beyond cross‐sectional survey approaches. On this basis, we can say with some confidence that we would expect that the coping ability of neighbourhoods across the UK to reflect their pre‐existing levels of community cohesion and identification, something already noted by commentators elsewhere (Felici, 2020; Tiratelli & Kaye, 2020). Specifically, communities high in identification and shared social support prior to the crisis should fare better than those which possess low levels of these qualities. Given the close association between deprivation and low levels of social capital, we can speculate that economically deprived neighbourhoods, marginalized, and stigmatized local areas will fare worst during the crisis.

This would be in part because of the concentration of individual and collective vulnerability factors in these areas. However, in addition, the low levels of infrastructure, resource, and support in these locales, along with sustained stigmatization and discrimination, will have corroded levels of community identification (McNamara et al., 2014). In such areas, the limited ability to help one’s neighbours is likely to limit the positive influence of shared norms of helping and of lockdown norm adherence. As national and local government and policy‐makers continue to rely on local communities to support vulnerable residents, they need to provide targeted support to local community‐based organizations, such as Mutual Aid groups. If such support can facilitate and foster the activities of these groups, it can help ensure that they are able to sustain their community‐based activities across the most disadvantaged communities, even in the face of escalating economic threat (Tiratelli & Kaye, 2020). Moreover, when lockdown restrictions ease, local authorities also increase their reliance on voluntary norm adherence among local community residents to control the virus spread (Prosser, Judge, Bolderdijk, Blackwood, & Kurz, 2020). Consequently, local community identity will become more, rather than less, important to sustain community‐protective behaviours after lockdown easing. This reinforces the point that local community identity and infrastructure needs to be fostered and developed by national and local authorities, in order to help communities to help themselves.

## Conflict of interest

All authors declare no conflict of interest.

## Supporting information


**Appendix S1**. All items of the community variables.

## Data Availability

The data that support the findings of this study are available from the corresponding author upon reasonable request.

## Footnotes

^1^
This research forms part of a three‐wave longitudinal study investigating the relationship between social psychological factors and resilience in residential settings. The first two waves were collected prior to COVID‐19, whereas the third took place during the lockdown and therefore offered the opportunity to explore how longstanding community identification shaped the response to the situation.

^2^
Due to perceived community support being conceptually similar to the giving and receiving of support during lockdown, confirmatory direct oblimin factor analysis was conducted. Results showed that i) T2 perceived community support was a distinct construct from T3 giving and receiving support during lockdown, and ii) T3 perceived community support was a distinct construct from T3 giving and receiving support during lockdown.
