# Supplementary material for: Collectively coping with coronavirus: Local community identification predicts giving support and lockdown adherence during the COVID‐19 pandemic
Source: Br J Soc Psychol. 2021 May 10;60(4):1403–18. doi: 10.1111/bjso.12457 (PMC8236966; doi:10.1111/bjso.12457)
Supplement: Supplementary file 1 — Appendix S1. All items of the community variables. [file BJSO-60-1403-s001.docx]

**Appendix A: All items of the community variables**

Community Identification

1. ‘I see myself as a member of my neighbourhood community.’
2. ‘I am pleased to be a member of my neighbourhood community.’
3. ‘I feel strong ties with members of my neighbourhood community.’
4. ‘I identify with other members of my neighbourhood community.’

Perceived Community Support T2

1. ‘I get the emotional support I need from other people in the neighbourhood.’
2. ‘I get the help I need from other people in the neighbourhood.’
3. ‘I get the resources I need from other people in the neighbourhood.’
4. ‘I get the advice I need from other people in the neighbourhood.’

Giving of Emotional Support during the Pandemic T3

1. ‘Gave emotional support to people in your local community.’
2. ‘Showed respect for others to people in your local community.’
3. ‘Showed concern for others’ needs to people in your local community.’

Receipt of Emotional Support during the Pandemic T3

1. ‘Given you emotional support.’
2. ‘Showed respect for you.’
3. ‘Showed concern for your needs.’
